# Crystal structures of Ca(ClO_4_)_2_·4H_2_O and Ca(ClO_4_)_2_·6H_2_O

**DOI:** 10.1107/S1600536814024532

**Published:** 2014-11-15

**Authors:** Erik Hennings, Horst Schmidt, Wolfgang Voigt

**Affiliations:** a TU Bergakademie Freiberg, Institute of Inorganic Chemistry, Leipziger Strasse 29, D-09596 Freiberg, Germany

**Keywords:** crystal structure, low-temperature salt hydrates, perchlorate hydrates, calcium salts, Mars minerals

## Abstract

The title compounds, calcium perchlorate tetra­hydrate and calcium perchlorate hexa­hydrate, were crystallized at low temperatures according to the solid–liquid phase diagram. The structure of the tetra­hydrate consists of one Ca^2+^ cation eightfold coordinated in a square-anti­prismatic fashion by four water mol­ecules and four O atoms of four perchlorate tetra­hedra, forming chains parallel to [01-1] by sharing corners of the ClO_4_ tetra­hedra. The structure of the hexa­hydrate contains two different Ca^2+^ cations, each coordinated by six water mol­ecules and two O atoms of two perchlorate tetra­hedra, forming [Ca(H_2_O)_6_(ClO_4_)]_2_ dimers by sharing two ClO_4_ tetra­hedra. The dimers are arranged in sheets parallel (001) and alternate with layers of non-coordinating ClO_4_ tetra­hedra. O—H⋯O hydrogen bonds between the water mol­ecules as donor and ClO_4_ tetra­hedra and water mol­ecules as acceptor groups lead to the formation of a three-dimensional network in the two structures. Ca(ClO_4_)_2_·6H_2_O was refined as a two-component inversion twin, with an approximate twin component ratio of 1:1 in each of the two structures.

## Abstract 2

The crystal structures of the tetra- and hexa­hydrate phases of Ca(ClO_4_)_2_ consist of Ca^2+^ ions in distorted square-anti­prismatic environments and of perchlorate tetra­hedra. O—H⋯O hydrogen bonds between water mol­ecules and ClO_4_ units lead to the formation of a three-dimensional network in the structures.

## Chemical context   

Since the detection of perchlorates on Mars during the Phoenix Mission (Chevrier *et al.*, 2009 ▶), inter­est in these salts, and especially their hydrates, has risen considerably (Kim *et al.*, 2013 ▶; Quinn *et al.*, 2013 ▶; Kerr, 2013 ▶; Davila *et al.*, 2013 ▶; Schuttlefield *et al.*, 2011 ▶; Navarro-González *et al.*, 2010 ▶; Marion *et al.*, 2010 ▶). To gain more knowledge about the behavior of salts and salt hydrates, it is essential to determine the corresponding phase diagrams. For calcium perchlorate, this was performed by several authors (Marion *et al.*, 2010 ▶; Pestova *et al.*, 2005 ▶; Dobrynina, 1984 ▶; Lilich & Djurinskii, 1956 ▶; Nicholson & Felsing, 1950 ▶; Willard & Smith, 1923 ▶) for different concentration areas with different results. The stable salt hydrate phase at room temperature in this system is calcium perchlorate tetra­hydrate. At lower temperatures, a higher hydrated phase, *i.e.* the hexa­hydrate, occurs as the stable phase.

## Structural commentary   

**Figure 1:**
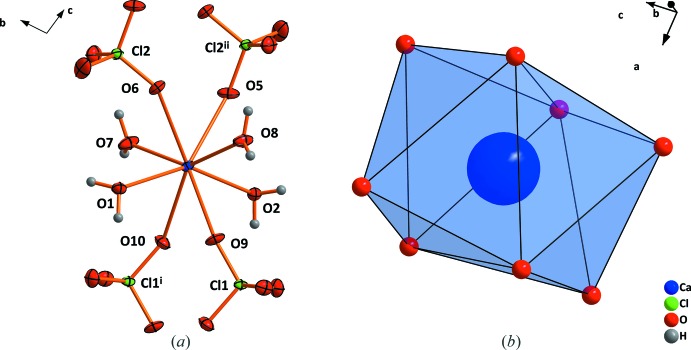
(*a*) The principle building block in the structure of Ca(ClO_4_)_2_·4H_2_O and (*b*) the square anti-prismatic coordination of Ca^2+^. Displacement ellipsoids are drawn at the 50% probability level. [Symmetry codes: (i) 1 − *x*, −*y*, 1 − *z*; (ii) 1 − *x*, 1 − *y*, 2 − *z*.]

The Ca^2+^ cation in Ca(ClO_4_)·4H_2_O is coordinated by four water mol­ecules (O1, O2, O7, O8) and four O atoms from two pairs of symmetry-related perchlorate tetra­hedra as shown in Fig. 1 ▶ *a*. The resulting coordination polyhedron is a distorted square anti-prism (Fig. 1 ▶ *b*). The Ca—O bond lengths involving the water mol­ecules range from 2.3284 (17) to 2.4153 (16) Å and are considerably shorter than the Ca—O bond lengths involving the perchlorate O atoms [2.5417 (16) to 2.5695 (17) Å].

**Figure 2:**
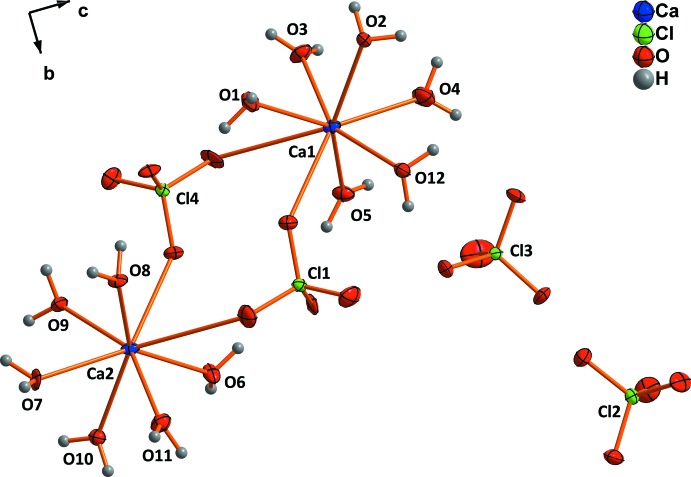
The principle building blocks in the structure of Ca(ClO_4_)_2_·6H_2_O. Displacement ellipsoids are drawn at the 50% probability level.

The two different Ca^2+^ cations in Ca(ClO_4_)·6H_2_O are each coordinated by six water mol­ecules and two perchlorate tetra­hedra (Fig. 2 ▶). Again, the bond lengths between the cations and water mol­ecules [2.319 (6)–2.500 (6) Å] are shorter than those to the perchlorate groups. For the latter, one of the two distances for each of the Ca^2+^ cations is by 0.5 Å markedly longer than the other (∼3.07 *versus* ∼2.53 Å). Nevertheless, according to the bond-valence model (Brown, 2002 ▶), the longer bond contributes *ca*. 0.05 valence units to the overall bond-valence sum and hence should not be neglected. If this longer bond is considered to be relevant, again a square anti-prismatic coordination polyhedron is realised for both Ca^2+^ cations, however with a much greater distortion. Two perchlorate tetra­hedra in the hexa­hydrate are shared between two Ca^2+^ ions, leading to the formation of [Ca(H_2_O)_6_(ClO_4_)]_2_ dimers oriented in layers parallel to (001).

## Supra­molecular features   

**Figure 3:**
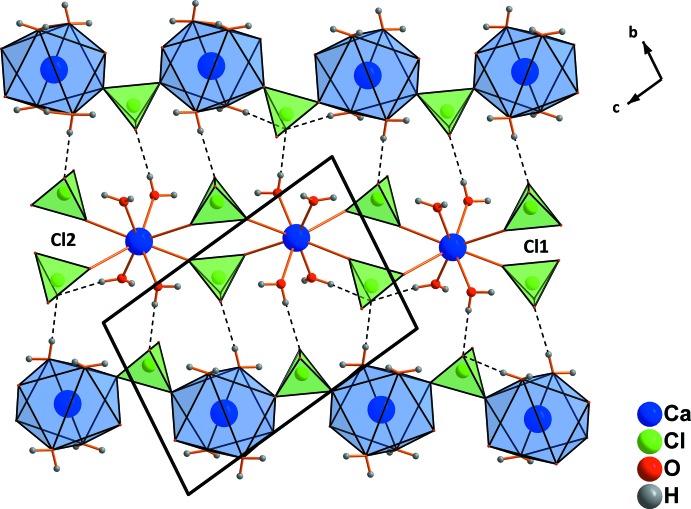
Formation of sheets and inter­connection of chains *via* hydrogen bonds in Ca(ClO_4_)_2_·4H_2_O. Only the strongest hydrogen bonds are shown, represented by dashed lines.

The perchlorate tetra­hedra in the structure of Ca(ClO_4_)·4H_2_O are shared between two adjacent Ca^2+^ ions, forming chains extending parallel to [01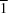] (Fig. 3 ▶) whereby each Ca^2+^ ion is connected along the chain on one side with a pair of Cl1 perchlorate tetra­hedra, and on the opposite side with a pair of Cl2 perchlorate tetra­hedra. The chains are arranged in sheets parallel to (0
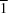
1) and are linked by O—H⋯O hydrogen bonds into a three-dimensional network with similar O⋯O distances between the water mol­ecules and the perchlorate tetra­hedra (Table 1 ▶).

**Figure 4:**
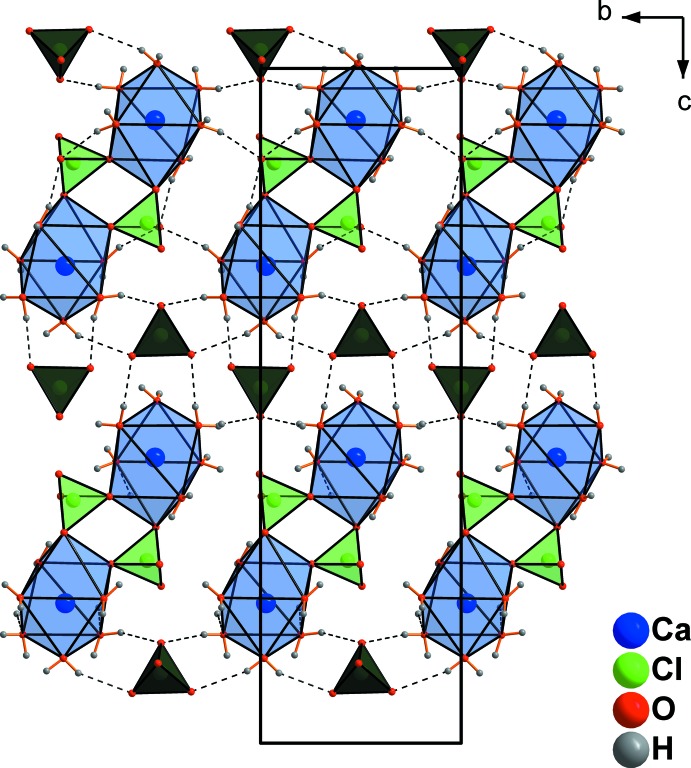
Formation of perchlorate-bridged dimers in Ca(ClO_4_)_2_·6H_2_O and location of ‘free’ perchlorate tetra­hedra in the gaps between the dimers (highlighted in dark green). Only the strongest hydrogen bonds are shown, represented by dashed lines.

**Figure 5:**
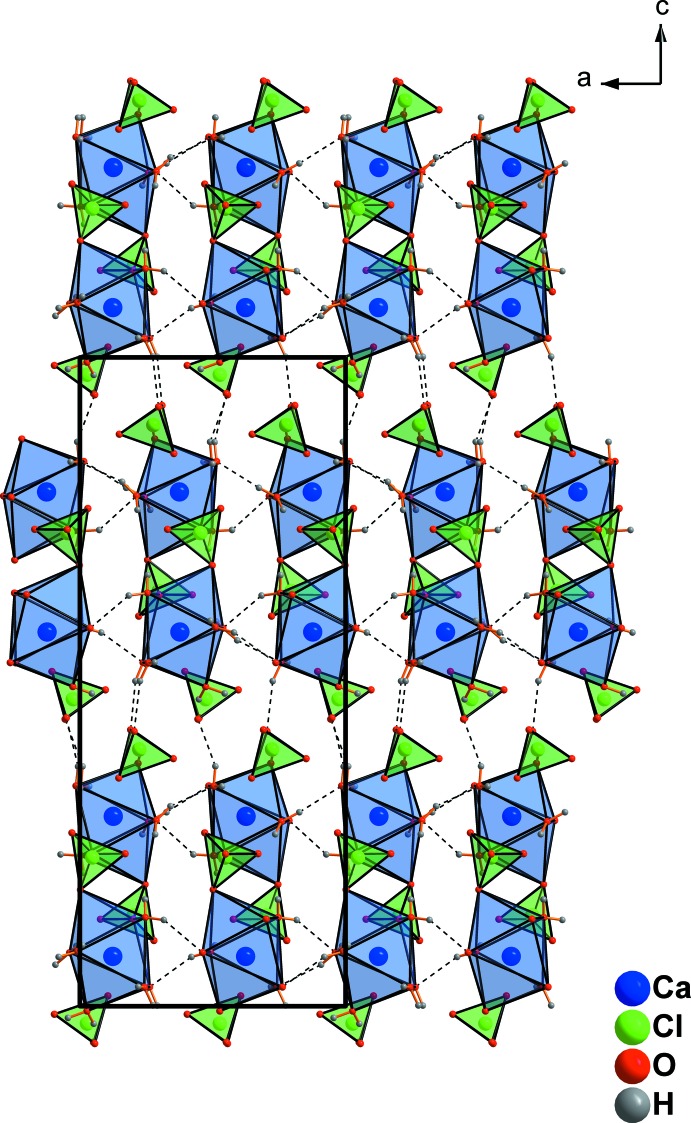
Formation of layers parallel to (001) in Ca(ClO_4_)_2_·6H_2_O. Only the strongest hydrogen bonds are shown, represented by dashed lines.

In addition to the two coordinating perchlorate tetra­hedra in Ca(ClO_4_)·6H_2_O, two ‘free’ perchlorate tetra­hedra are present in the crystal structure. These ‘free’ ClO_4_ tetra­hedra are arranged in sheets and alternate with the [Ca(H_2_O)_6_(ClO_4_)]_2_ sheets along [001] (Fig. 4 ▶). The ‘free’ perchlorate tetra­hedra are connected to the dimers *via* O—H⋯O hydrogen bonds, as shown in Fig. 4 ▶. The dimers are additionally connected through further O—H⋯O hydrogen bonds (Table 2 ▶) into a three-dimensional network (Fig. 5 ▶).

## Database survey   

For crystal structures of other *M(*ClO_4_)_2_·4H_2_O phases, see: Robertson & Bish (2010 ▶; *M* = Mg); Hennings *et al.* (2014 ▶; Sr); Solovyov (2012 ▶; Mg); Johansson (1966 ▶; Hg). For crystal structures of other *M(*ClO_4_)_2_·6H_2_O phases, see: Ghosh *et al.* (1997 ▶; *M* = Ni, Zn); Ghosh & Ray (1981 ▶; Fe); Johansson *et al.* (1978 ▶; Hg); Mani & Ramaseshan (1961 ▶; Cu); Johansson & Sandström (1987 ▶; Cd); Gallucci & Gerkin (1989 ▶; Cu); West (1935 ▶; Mg).

## Synthesis and crystallization   

Ca(ClO_4_)_2_·4H_2_O was crystallized from an aqueous solution of 62.96 wt% Ca(ClO_4_)_2_ at 273 K after one day and Ca(ClO_4_)_2_·6H_2_O from an aqueous solution of 57.55 wt% Ca(ClO_4_)_2_ at 238 K after one week. For the preparation of these aqueous solutions, Ca(ClO_4_)_2_·4H_2_O (Acros Organics, p.A.) was used. The Ca^2+^ content was analysed *via* complexometric titration with EDTA. The crystals remain stable in the saturated aqueous solution over at least four weeks.

The samples were stored in a freezer or a cryostat at low temperatures. The crystals were separated and embedded in perfluorinated ether for X-ray analysis.

## Refinement   

Crystal data, data collection and structure refinement details are summarized in Table 3 ▶. The H atoms of each structure were placed in the positions indicated by difference Fourier maps. For Ca(ClO_4_)_2_·4H_2_O, distance restraints were applied for all water mol­ecules, with O—H and H—H distance restraints of 0.82 (1) and 1.32 (1) Å, respectively. For Ca(ClO_4_)_2_·6H_2_O, *U*_iso_ values were set at 1.2*U*_eq_(O) using a riding-model approximation. Distance restraints were applied for that structure for all water mol­ecules, with O—H and H—H distance restraints of 0.84 (2) and 1.4 (2) Å, respectively. Ca(ClO_4_)_2_·6H_2_O was refined as a two-component inversion twin, with an approximate twin component ratio of 1:1.

## Supplementary Material

Crystal structure: contains datablock(s) CaClO4_4H2O_200K, CaClO4_6H2O_180K. DOI: 10.1107/S1600536814024532/wm5079sup1.cif

Structure factors: contains datablock(s) CaClO4_4H2O_200K. DOI: 10.1107/S1600536814024532/wm5079CaClO4_4H2O_200Ksup2.hkl

Structure factors: contains datablock(s) CaClO4_6H2O_180K. DOI: 10.1107/S1600536814024532/wm5079CaClO4_6H2O_180Ksup3.hkl

Supporting information file. DOI: 10.1107/S1600536814024532/wm5079CaClO4_4H2O_200Ksup4.cml

Supporting information file. DOI: 10.1107/S1600536814024532/wm5079CaClO4_6H2O_180Ksup5.cml

CCDC references: 1033323, 1033324

Additional supporting information:  crystallographic information; 3D view; checkCIF report

## Figures and Tables

**Table 1:** Hydrogen-bond geometry (, ) for Ca(ClO_4_)_2_4H_2_O

*D*H*A*	*D*H	H*A*	*D* *A*	*D*H*A*
O1H1*B*O11^i^	0.82(1)	2.11(2)	2.888(2)	158(3)
O1H1*A*O3^ii^	0.82(1)	2.13(1)	2.947(2)	174(3)
O2H2*A*O11^iii^	0.82(1)	2.17(2)	2.947(2)	159(3)
O2H2*B*O4^iv^	0.82(1)	2.02(1)	2.830(2)	172(3)
O7H7*B*O4	0.81(1)	2.22(2)	2.924(2)	146(3)
O7H7*A*O1^iii^	0.82(1)	2.06(1)	2.870(2)	172(3)
O8H8*A*O4^v^	0.82(1)	2.33(3)	2.986(2)	137(4)
O8H8*B*O2^vi^	0.82(1)	2.14(1)	2.950(2)	169(5)

Symmetry codes: (i) 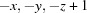; (ii) 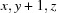; (iii) 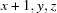; (iv) 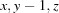; (v) 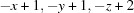; (vi) 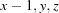.

**Table 2:** Hydrogen-bond geometry (, ) for Ca(ClO_4_)_2_6H_2_O

*D*H*A*	*D*H	H*A*	*D* *A*	*D*H*A*
O1H1*A*O15	0.84(2)	2.07(3)	2.887(10)	164(8)
O1H1*B*O5^i^	0.84(2)	2.25(5)	2.915(7)	136(6)
O1H1*B*O16^i^	0.84(2)	2.44(5)	3.132(10)	140(6)
O2H2*A*O23^ii^	0.84(2)	2.03(2)	2.856(9)	169(7)
O2H2*B*O26^iii^	0.84(2)	2.14(3)	2.932(8)	155(6)
O3H3*A*O12^iv^	0.84(2)	2.07(2)	2.899(8)	168(8)
O3H3*B*O19^iii^	0.84(2)	2.15(3)	2.934(8)	156(7)
O4H4*A*O27	0.84(2)	2.28(3)	3.074(11)	158(8)
O4H4*B*O28^iii^	0.84(2)	2.36(3)	3.177(10)	163(8)
O5H5*A*O2^iv^	0.84(2)	1.98(3)	2.783(8)	159(7)
O5H5*B*O19	0.84(2)	2.20(5)	2.903(9)	142(6)
O6H6*A*O8^v^	0.84(2)	2.18(4)	2.925(7)	149(7)
O6H6*B*O19	0.84(2)	2.08(3)	2.891(10)	162(8)
O7H7*A*O23^vi^	0.84(2)	2.29(4)	3.042(9)	149(6)
O7H7*B*O24^vii^	0.84(2)	2.50(5)	3.199(9)	141(6)
O7H7*B*O27^viii^	0.84(2)	2.57(5)	3.242(11)	138(6)
O8H8*A*O10^ix^	0.84(2)	2.08(4)	2.805(8)	145(6)
O8H8*B*O15	0.84(2)	2.07(3)	2.879(9)	162(7)
O9H9*A*O27^x^	0.84(2)	2.06(3)	2.865(10)	161(7)
O9H9*B*O21^vi^	0.84(2)	2.23(5)	2.962(10)	145(7)
O10H10*A*O21^vii^	0.84(2)	2.12(3)	2.930(9)	163(7)
O10H10*B*O28^x^	0.84(2)	2.10(3)	2.902(10)	162(7)
O11H11*A*O9^ix^	0.84(2)	2.14(4)	2.893(9)	150(7)
O11H11*B*O15^xi^	0.84(2)	2.11(3)	2.915(9)	161(7)
O12H12*A*O26	0.84(2)	2.35(5)	2.995(9)	135(6)
O12H12*A*O20	0.84(2)	2.40(4)	3.102(9)	142(6)
O12H12*B*O24^ii^	0.84(2)	2.03(2)	2.861(9)	171(7)

Symmetry codes: (i) 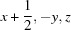; (ii) 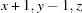; (iii) 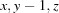; (iv) 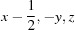; (v) 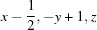; (vi) 
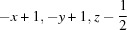
; (vii) 
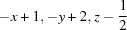
; (viii) 
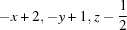
; (ix) 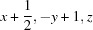; (x) 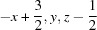; (xi) 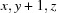.

**Table 3:** Experimental details

	Ca(ClO_4_)_2_4H_2_O	Ca(ClO_4_)_2_6H_2_O
Crystal data		
*M*_r_	311.04	347.08
Crystal system, space group	Triclinic, *P* 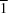	Orthorhombic, *P* *c* *a*2_1_
Temperature (K)	200	180
*a*, *b*, *c* ()	5.4886(11), 7.8518(15), 11.574(2)	10.9603(4), 7.9667(7), 26.7735(18)
, , ()	99.663(16), 90.366(16), 90.244(16)	90, 90, 90
*V* (^3^)	491.71(17)	2337.8(3)
*Z*	2	8
Radiation type	Mo *K*	Mo *K*
(mm^1^)	1.24	1.06
Crystal size (mm)	0.04 0.03 0.02	0.38 0.31 0.08

Data collection		
Diffractometer	Stoe IPDS2	Stoe IPDS2
Absorption correction	Integration Coppens (1970 ▶)	Integration (Coppens, 1970 ▶)
*T*_min_, *T*_max_	0.644, 0.789	0.684, 0.923
No. of measured, independent and observed [*I* > 2(*I*)] reflections	2659, 2636, 2529	15755, 5326, 4919
*R*_int_	0.074	0.062
(sin /)_max_ (^1^)	0.686	0.650

Refinement		
*R*[*F* ^2^ > 2(*F* ^2^)], *wR*(*F* ^2^), *S*	0.031, 0.089, 1.20	0.042, 0.113, 1.09
No. of reflections	2636	5326
No. of parameters	168	380
No. of restraints	12	37
H-atom treatment	All H-atom parameters refined	Only H-atom coordinates refined
_max_, _min_ (e ^3^)	0.36, 0.75	0.41, 0.67
Absolute structure		Refined as an inversion twin
Absolute structure parameter		0.45(9)

Computer programs: *X-AREA* and *X-RED* (Stoe Cie, 2009 ▶), *SHELXS97* and *SHELXL2012* (Sheldrick, 2008 ▶), *DIAMOND* (Brandenburg, 2006 ▶) and *publCIF* (Westrip, 2010 ▶).

## supplementary crystallographic information

### Crystal data

**Table:**

Ca(ClO_4_)_2_·6H_2_O	*D*_x_ = 1.972 Mg m^−^^3^
*M**_r_* = 347.08	Mo *K*α radiation, λ = 0.71073 Å
Orthorhombic, *P**c**a*2_1_	Cell parameters from 17254 reflections
*a* = 10.9603 (4) Å	θ = 2.9–29.6°
*b* = 7.9667 (7) Å	µ = 1.06 mm^−^^1^
*c* = 26.7735 (18) Å	*T* = 180 K
*V* = 2337.8 (3) Å^3^	Plate, colourless
*Z* = 8	0.38 × 0.31 × 0.08 mm
*F*(000) = 1424	

### Data collection

**Table:**

Stoe IPDS-2 diffractometer	5326 independent reflections
Radiation source: fine-focus sealed tube	4919 reflections with *I* > 2σ(*I*)
Graphite monochromator	*R*_int_ = 0.062
Detector resolution: 6.67 pixels mm^-1^	θ_max_ = 27.5°, θ_min_ = 1.5°
rotation method scans	*h* = −15→15
Absorption correction: integration (Coppens, 1970)	*k* = −11→9
*T*_min_ = 0.684, *T*_max_ = 0.923	*l* = −37→37
15755 measured reflections	

### Refinement

**Table:**

Refinement on *F*^2^	Hydrogen site location: difference Fourier map
Least-squares matrix: full	Only H-atom coordinates refined
*R*[*F*^2^ > 2σ(*F*^2^)] = 0.042	*w* = 1/[σ^2^(*F*_o_^2^) + (0.0687*P*)^2^ + 2.3411*P*] where *P* = (*F*_o_^2^ + 2*F*_c_^2^)/3
*wR*(*F*^2^) = 0.113	(Δ/σ)_max_ < 0.001
*S* = 1.09	Δρ_max_ = 0.41 e Å^−^^3^
5326 reflections	Δρ_min_ = −0.67 e Å^−^^3^
380 parameters	Absolute structure: Refined as an inversion twin
37 restraints	Absolute structure parameter: 0.45 (9)

### Special details

**Table:**

Geometry. All esds (except the esd in the dihedral angle between two l.s. planes) are estimated using the full covariance matrix. The cell esds are taken into account individually in the estimation of esds in distances, angles and torsion angles; correlations between esds in cell parameters are only used when they are defined by crystal symmetry. An approximate (isotropic) treatment of cell esds is used for estimating esds involving l.s. planes.
Refinement. Refined as a 2-component inversion twin.

### Fractional atomic coordinates and isotropic or equivalent isotropic displacement parameters (Å^2^)

**Table:**

	*x*	*y*	*z*	*U*_iso_*/*U*_eq_	
Ca1	0.87471 (15)	0.02736 (19)	0.29261 (6)	0.0110 (3)	
Ca2	0.87640 (16)	0.47462 (18)	0.07672 (6)	0.0112 (3)	
Cl3	0.7770 (2)	0.50239 (14)	0.39582 (8)	0.0102 (5)	
Cl4	0.79753 (11)	0.06649 (15)	0.13985 (8)	0.0110 (2)	
Cl1	0.95348 (11)	0.43473 (15)	0.22917 (8)	0.0109 (2)	
Cl2	0.0260 (2)	0.99991 (14)	0.47324 (8)	0.0128 (5)	
O5	0.7128 (5)	0.2189 (8)	0.2859 (2)	0.0169 (11)	
H5B	0.714 (6)	0.295 (6)	0.264 (2)	0.020*	
H5A	0.660 (5)	0.209 (10)	0.3081 (19)	0.020*	
O3	0.7416 (5)	−0.1972 (7)	0.2841 (2)	0.0185 (12)	
H3A	0.672 (3)	−0.190 (10)	0.296 (3)	0.022*	
H3B	0.752 (7)	−0.274 (6)	0.263 (2)	0.022*	
O7	0.9726 (7)	0.4844 (6)	−0.0075 (3)	0.0162 (14)	
H7B	0.995 (7)	0.557 (7)	−0.028 (2)	0.019*	
H7A	0.989 (6)	0.406 (6)	−0.027 (2)	0.019*	
O8	1.0380 (5)	0.2796 (7)	0.0823 (2)	0.0132 (10)	
H8A	1.094 (4)	0.245 (8)	0.064 (2)	0.016*	
H8B	1.013 (6)	0.186 (4)	0.093 (2)	0.016*	
O6	0.7539 (4)	0.6064 (6)	0.13676 (16)	0.0190 (8)	
H6B	0.761 (7)	0.565 (10)	0.1655 (14)	0.023*	
H6A	0.684 (3)	0.648 (9)	0.133 (3)	0.023*	
O1	0.9967 (4)	−0.1049 (6)	0.23219 (17)	0.0186 (8)	
H1A	0.987 (7)	−0.060 (10)	0.2041 (15)	0.022*	
H1B	1.0726 (17)	−0.118 (9)	0.232 (3)	0.022*	
O2	0.9964 (5)	−0.1849 (7)	0.3393 (2)	0.0119 (11)	
H2A	1.007 (7)	−0.184 (9)	0.3702 (7)	0.014*	
H2B	0.967 (6)	−0.281 (4)	0.335 (2)	0.014*	
O4	0.7803 (9)	0.0171 (8)	0.3739 (3)	0.0262 (18)	
H4B	0.793 (8)	−0.068 (6)	0.392 (3)	0.031*	
H4A	0.795 (7)	0.093 (7)	0.395 (2)	0.031*	
O15	0.9234 (8)	0.0045 (5)	0.1339 (4)	0.0152 (16)	
O14	0.7230 (8)	−0.0046 (5)	0.1019 (3)	0.0194 (18)	
O19	0.8302 (8)	0.4971 (5)	0.2349 (3)	0.0142 (15)	
O20	1.0316 (9)	0.5052 (6)	0.2678 (4)	0.027 (2)	
O26	0.8567 (9)	0.5044 (5)	0.3525 (3)	0.0215 (19)	
O12	1.0148 (5)	0.2008 (7)	0.3398 (2)	0.0129 (11)	
H12B	1.034 (7)	0.189 (9)	0.3699 (10)	0.016*	
H12A	1.006 (6)	0.303 (3)	0.334 (2)	0.016*	
O16	0.7540 (9)	0.0215 (7)	0.1877 (3)	0.0234 (15)	
O21	0.1020 (10)	0.9989 (6)	0.5168 (3)	0.026 (2)	
O28	0.7947 (7)	0.6558 (9)	0.4237 (3)	0.0231 (15)	
O27	0.8080 (7)	0.3589 (9)	0.4260 (3)	0.0260 (16)	
O23	0.0566 (6)	0.8560 (8)	0.4423 (3)	0.0183 (13)	
O24	0.0519 (7)	1.1496 (9)	0.4444 (3)	0.0212 (14)	
O18	0.9975 (9)	0.4783 (7)	0.1797 (3)	0.0223 (14)	
O13	0.7991 (3)	0.2473 (5)	0.13424 (18)	0.0150 (8)	
O17	0.9520 (4)	0.2544 (5)	0.23441 (19)	0.0158 (8)	
O9	0.7381 (6)	0.2976 (8)	0.0297 (2)	0.0171 (12)	
H9A	0.721 (7)	0.338 (8)	0.0016 (13)	0.021*	
H9B	0.753 (7)	0.195 (3)	0.025 (3)	0.021*	
O22	−0.0976 (9)	0.9933 (7)	0.4886 (5)	0.034 (2)	
O25	0.6508 (8)	0.4900 (7)	0.3824 (5)	0.034 (2)	
O10	0.7580 (5)	0.6907 (7)	0.0295 (2)	0.0139 (11)	
H10B	0.746 (7)	0.658 (8)	0.0002 (11)	0.017*	
H10A	0.783 (6)	0.789 (4)	0.025 (2)	0.017*	
O11	1.0097 (5)	0.7020 (8)	0.0838 (3)	0.0192 (12)	
H11A	1.079 (3)	0.736 (9)	0.075 (3)	0.023*	
H11B	0.987 (7)	0.799 (4)	0.092 (3)	0.023*	

### Atomic displacement parameters (Å^2^)

**Table:**

	*U*^11^	*U*^22^	*U*^33^	*U*^12^	*U*^13^	*U*^23^
Ca1	0.0083 (6)	0.0090 (5)	0.0157 (7)	0.0010 (5)	0.0018 (4)	−0.0005 (7)
Ca2	0.0095 (6)	0.0084 (5)	0.0155 (7)	0.0010 (5)	0.0021 (4)	0.0016 (7)
Cl3	0.0096 (11)	0.0097 (11)	0.0113 (11)	−0.0005 (4)	0.0016 (10)	0.0004 (4)
Cl4	0.0127 (5)	0.0095 (6)	0.0108 (5)	−0.0007 (4)	0.0020 (4)	0.0012 (5)
Cl1	0.0124 (6)	0.0080 (6)	0.0122 (5)	−0.0018 (4)	0.0019 (4)	0.0008 (5)
Cl2	0.0180 (13)	0.0098 (11)	0.0105 (11)	−0.0001 (4)	0.0016 (11)	0.0002 (4)
O5	0.015 (2)	0.015 (2)	0.020 (2)	0.0012 (19)	0.0022 (19)	0.0054 (19)
O3	0.019 (3)	0.013 (2)	0.024 (2)	−0.007 (2)	0.007 (2)	−0.0047 (18)
O7	0.022 (3)	0.019 (3)	0.008 (3)	0.0018 (18)	0.009 (2)	−0.0048 (16)
O8	0.011 (2)	0.0077 (19)	0.020 (2)	0.0033 (17)	0.0061 (18)	−0.0006 (17)
O6	0.0201 (19)	0.024 (2)	0.0130 (16)	0.0123 (17)	0.0008 (17)	0.0029 (18)
O1	0.0196 (19)	0.021 (2)	0.0151 (17)	0.0079 (17)	0.0003 (17)	0.0028 (18)
O2	0.017 (2)	0.009 (2)	0.009 (2)	−0.0002 (19)	−0.0009 (17)	0.0010 (17)
O4	0.033 (4)	0.023 (3)	0.023 (4)	0.000 (2)	0.008 (3)	0.006 (2)
O15	0.014 (4)	0.010 (3)	0.022 (4)	0.0049 (13)	−0.001 (3)	−0.0002 (14)
O14	0.019 (4)	0.019 (4)	0.021 (4)	−0.0067 (15)	0.000 (3)	−0.0045 (15)
O19	0.013 (4)	0.019 (3)	0.011 (3)	0.0049 (14)	0.007 (3)	0.0011 (13)
O20	0.027 (5)	0.023 (4)	0.033 (5)	−0.0106 (18)	−0.016 (4)	−0.0050 (18)
O26	0.034 (5)	0.011 (3)	0.020 (4)	−0.0004 (16)	0.014 (4)	0.0002 (14)
O12	0.016 (2)	0.011 (2)	0.013 (2)	0.0011 (19)	−0.0010 (17)	0.0021 (18)
O16	0.027 (3)	0.022 (2)	0.021 (3)	0.001 (2)	0.015 (2)	0.011 (2)
O21	0.035 (5)	0.029 (4)	0.013 (4)	0.0009 (18)	−0.010 (4)	0.0018 (15)
O28	0.037 (4)	0.011 (3)	0.021 (3)	−0.004 (2)	0.002 (3)	−0.005 (2)
O27	0.042 (4)	0.017 (3)	0.019 (3)	0.008 (3)	0.003 (3)	0.008 (3)
O23	0.027 (3)	0.014 (3)	0.015 (3)	0.002 (2)	0.001 (2)	−0.006 (2)
O24	0.034 (3)	0.012 (3)	0.017 (3)	−0.003 (2)	−0.006 (2)	0.008 (2)
O18	0.025 (3)	0.027 (2)	0.015 (3)	0.001 (2)	0.012 (2)	0.002 (2)
O13	0.0195 (19)	0.0081 (18)	0.017 (2)	0.0011 (14)	0.0007 (15)	0.0020 (15)
O17	0.0197 (19)	0.0074 (17)	0.020 (2)	−0.0009 (15)	−0.0007 (15)	0.0014 (16)
O9	0.025 (3)	0.011 (2)	0.016 (2)	−0.003 (2)	−0.003 (2)	0.0006 (19)
O22	0.024 (5)	0.032 (4)	0.044 (6)	0.0013 (19)	0.013 (4)	0.000 (2)
O25	0.010 (4)	0.038 (4)	0.054 (6)	−0.0011 (19)	−0.014 (4)	0.004 (2)
O10	0.012 (2)	0.014 (2)	0.016 (2)	0.0008 (19)	0.0021 (18)	0.0005 (18)
O11	0.016 (3)	0.015 (2)	0.027 (2)	−0.002 (2)	0.000 (2)	−0.0030 (19)

### Geometric parameters (Å, º)

**Table:**

Ca1—O3	2.319 (6)	Cl3—O25	1.432 (9)
Ca1—O5	2.347 (6)	Cl3—O27	1.440 (7)
Ca1—O1	2.349 (5)	Cl3—O28	1.446 (7)
Ca1—O4	2.412 (9)	Cl3—O26	1.452 (9)
Ca1—O12	2.421 (6)	Cl4—O16	1.414 (8)
Ca1—O2	2.490 (6)	Cl4—O14	1.421 (8)
Ca1—O17	2.533 (5)	Cl4—O13	1.449 (4)
Ca1—O16	3.104 (9)	Cl4—O15	1.474 (8)
Ca2—O11	2.335 (6)	Cl1—O17	1.444 (4)
Ca2—O6	2.343 (5)	Cl1—O19	1.448 (8)
Ca2—O8	2.360 (5)	Cl1—O18	1.451 (8)
Ca2—O9	2.423 (6)	Cl1—O20	1.455 (9)
Ca2—O7	2.491 (7)	Cl2—O22	1.416 (10)
Ca2—O10	2.500 (6)	Cl2—O21	1.433 (10)
Ca2—O13	2.523 (4)	Cl2—O24	1.449 (7)
Ca2—O18	3.061 (9)	Cl2—O23	1.453 (7)
			
O3—Ca1—O5	91.1 (3)	O7—Ca2—O10	74.9 (2)
O3—Ca1—O1	86.81 (19)	O11—Ca2—O13	135.8 (2)
O5—Ca1—O1	132.0 (2)	O6—Ca2—O13	73.17 (15)
O3—Ca1—O4	78.0 (2)	O8—Ca2—O13	75.00 (17)
O5—Ca1—O4	76.5 (3)	O9—Ca2—O13	71.91 (17)
O1—Ca1—O4	148.3 (2)	O7—Ca2—O13	135.81 (17)
O3—Ca1—O12	152.9 (2)	O10—Ca2—O13	128.95 (17)
O5—Ca1—O12	98.5 (2)	O11—Ca2—O18	69.4 (2)
O1—Ca1—O12	104.72 (19)	O6—Ca2—O18	68.03 (19)
O4—Ca1—O12	79.7 (3)	O8—Ca2—O18	67.9 (2)
O3—Ca1—O2	82.1 (2)	O9—Ca2—O18	138.2 (2)
O5—Ca1—O2	152.3 (2)	O7—Ca2—O18	129.2 (3)
O1—Ca1—O2	74.68 (18)	O10—Ca2—O18	132.42 (19)
O4—Ca1—O2	75.8 (2)	O13—Ca2—O18	66.58 (17)
O12—Ca1—O2	77.7 (2)	O25—Cl3—O27	108.3 (5)
O3—Ca1—O17	134.5 (2)	O25—Cl3—O28	108.5 (5)
O5—Ca1—O17	75.01 (18)	O27—Cl3—O28	110.4 (5)
O1—Ca1—O17	72.92 (15)	O25—Cl3—O26	112.4 (7)
O4—Ca1—O17	136.29 (19)	O27—Cl3—O26	108.3 (4)
O12—Ca1—O17	72.61 (17)	O28—Cl3—O26	108.8 (4)
O2—Ca1—O17	127.91 (18)	O16—Cl4—O14	110.7 (5)
O3—Ca1—O16	68.4 (2)	O16—Cl4—O13	110.5 (3)
O5—Ca1—O16	67.5 (2)	O14—Cl4—O13	109.2 (3)
O1—Ca1—O16	67.2 (2)	O16—Cl4—O15	109.2 (5)
O4—Ca1—O16	129.3 (3)	O14—Cl4—O15	109.1 (5)
O12—Ca1—O16	138.63 (19)	O13—Cl4—O15	108.1 (3)
O2—Ca1—O16	132.21 (19)	O17—Cl1—O19	108.7 (3)
O17—Ca1—O16	66.22 (16)	O17—Cl1—O18	109.3 (3)
O11—Ca2—O6	87.4 (2)	O19—Cl1—O18	109.0 (5)
O11—Ca2—O8	92.1 (3)	O17—Cl1—O20	108.8 (3)
O6—Ca2—O8	133.0 (2)	O19—Cl1—O20	110.0 (5)
O11—Ca2—O9	152.3 (2)	O18—Cl1—O20	111.1 (5)
O6—Ca2—O9	105.0 (2)	O22—Cl2—O21	108.6 (7)
O8—Ca2—O9	96.8 (2)	O22—Cl2—O24	111.9 (4)
O11—Ca2—O7	77.6 (2)	O21—Cl2—O24	108.9 (4)
O6—Ca2—O7	148.20 (18)	O22—Cl2—O23	110.9 (4)
O8—Ca2—O7	76.1 (2)	O21—Cl2—O23	108.9 (4)
O9—Ca2—O7	79.2 (2)	O24—Cl2—O23	107.5 (5)
O11—Ca2—O10	80.3 (2)	Cl4—O16—Ca1	132.3 (5)
O6—Ca2—O10	75.00 (17)	Cl1—O18—Ca2	132.5 (5)
O8—Ca2—O10	151.0 (2)	Cl4—O13—Ca2	141.3 (3)
O9—Ca2—O10	79.2 (3)	Cl1—O17—Ca1	140.7 (3)

### Hydrogen-bond geometry (Å, º)

**Table:**

*D*—H···*A*	*D*—H	H···*A*	*D*···*A*	*D*—H···*A*
O1—H1*A*···O15	0.84 (2)	2.07 (3)	2.887 (10)	164 (8)
O1—H1*B*···O5^i^	0.84 (2)	2.25 (5)	2.915 (7)	136 (6)
O1—H1*B*···O16^i^	0.84 (2)	2.44 (5)	3.132 (10)	140 (6)
O2—H2*A*···O23^ii^	0.84 (2)	2.03 (2)	2.856 (9)	169 (7)
O2—H2*B*···O26^iii^	0.84 (2)	2.14 (3)	2.932 (8)	155 (6)
O3—H3*A*···O12^iv^	0.84 (2)	2.07 (2)	2.899 (8)	168 (8)
O3—H3*B*···O19^iii^	0.84 (2)	2.15 (3)	2.934 (8)	156 (7)
O4—H4*A*···O27	0.84 (2)	2.28 (3)	3.074 (11)	158 (8)
O4—H4*B*···O28^iii^	0.84 (2)	2.36 (3)	3.177 (10)	163 (8)
O5—H5*A*···O2^iv^	0.84 (2)	1.98 (3)	2.783 (8)	159 (7)
O5—H5*B*···O19	0.84 (2)	2.20 (5)	2.903 (9)	142 (6)
O6—H6*A*···O8^v^	0.84 (2)	2.18 (4)	2.925 (7)	149 (7)
O6—H6*B*···O19	0.84 (2)	2.08 (3)	2.891 (10)	162 (8)
O7—H7*A*···O23^vi^	0.84 (2)	2.29 (4)	3.042 (9)	149 (6)
O7—H7*B*···O24^vii^	0.84 (2)	2.50 (5)	3.199 (9)	141 (6)
O7—H7*B*···O27^viii^	0.84 (2)	2.57 (5)	3.242 (11)	138 (6)
O8—H8*A*···O10^ix^	0.84 (2)	2.08 (4)	2.805 (8)	145 (6)
O8—H8*B*···O15	0.84 (2)	2.07 (3)	2.879 (9)	162 (7)
O9—H9*A*···O27^x^	0.84 (2)	2.06 (3)	2.865 (10)	161 (7)
O9—H9*B*···O21^vi^	0.84 (2)	2.23 (5)	2.962 (10)	145 (7)
O10—H10*A*···O21^vii^	0.84 (2)	2.12 (3)	2.930 (9)	163 (7)
O10—H10*B*···O28^x^	0.84 (2)	2.10 (3)	2.902 (10)	162 (7)
O11—H11*A*···O9^ix^	0.84 (2)	2.14 (4)	2.893 (9)	150 (7)
O11—H11*B*···O15^xi^	0.84 (2)	2.11 (3)	2.915 (9)	161 (7)
O12—H12*A*···O26	0.84 (2)	2.35 (5)	2.995 (9)	135 (6)
O12—H12*A*···O20	0.84 (2)	2.40 (4)	3.102 (9)	142 (6)
O12—H12*B*···O24^ii^	0.84 (2)	2.03 (2)	2.861 (9)	171 (7)

Symmetry codes: (i) *x*+1/2, −*y*, *z*; (ii) *x*+1, *y*−1, *z*; (iii) *x*, *y*−1, *z*; (iv) *x*−1/2, −*y*, *z*; (v) *x*−1/2, −*y*+1, *z*; (vi) −*x*+1, −*y*+1, *z*−1/2; (vii) −*x*+1, −*y*+2, *z*−1/2; (viii) −*x*+2, −*y*+1, *z*−1/2; (ix) *x*+1/2, −*y*+1, *z*; (x) −*x*+3/2, *y*, *z*−1/2; (xi) *x*, *y*+1, *z*.
